# A New Classification Method for High-Volume Fly Ash: Performance Based on Coal Source and Particle Size

**DOI:** 10.3390/ma18174145

**Published:** 2025-09-04

**Authors:** Xiangnan Ji, Chen Zhang, Yaru Yang, Jiahao Zhang, Lin Tang, Dongxu Ji

**Affiliations:** 1 Development Research Center for Natural Resources and Real Estate Assessment Shenzhen, Shenzhen 518000, China; m15822762154@163.com (X.J.); tanglin202508@163.com (L.T.); 2 School of Maritime Law and Traffic Management, Guangzhou Maritime University, Guangzhou 510725, China; 3 Department of Architecture, Faculty of Environmental Engineering, The University of Kitakyushu, Kitakyushu 808-0135, Japan; e3dbb418@eng.kitakyu-u.ac.jp; 4 Faculty of Civil and Environmental Engineering, Harbin Institute of Technology, Shenzhen 518055, China; zjh773625249@gmail.com; 5 School of Science and Engineering, The Chinese University of Hong Kong, Shenzhen 518172, China; jidongxu@cuhk.edu.cn

**Keywords:** particle size, fly ash, flotation classification, thermal analysis, XRD, pozzolanic reactions

## Abstract

This study investigates the impact of particle size in fly ash derived from different coal sources on the performance of fly ash–cement systems. Utilizing a newly developed flotation classification method, physical properties of fly ash were examined to reveal variations among different particle sizes and coal sources. Thermal analysis was employed to analyze the calcium hydroxide content’s effect on the cement system, while selective dissolution methods were used to assess reaction rates. XRD analysis confirmed particle size effects. Results indicate that flotation classification optimizes the properties of fly ash, enhancing activity and flow values, where some of the ash fractions exhibit overall superior properties. The use of high-volume fly ash (50% fly ash replacement) promotes continued pozzolanic reactions, especially with smaller particle sizes. Reaction rates decrease with larger particle sizes, emphasizing the importance of classification. XRD analysis further supports these findings, revealing that smaller particle sizes favor cement hydration and pozzolanic reactions. Overall, this study provides insights into optimizing fly ash properties for enhanced concrete performance.

## 1. Introduction

As one of the primary global energy sources, coal is expected to maintain its critical role in energy supply for the foreseeable future [1]. Following the Fukushima nuclear disaster in 2011, Japan experienced a major restructuring of its energy mix [2]. The contribution of nuclear power to the national energy supply has markedly decreased, leading to a sustained expansion in coal-fired electricity generation to compensate for the energy deficit [3]. Consequently, the output of fly ash—a by-product of coal combustion—has risen significantly [4]. Storing large quantities of fly ash not only occupies limited land resources but also presents potential environmental hazards, such as contamination of water systems, air, and soil [5,6]. Nevertheless, from the standpoint of resource recovery and reuse, fly ash demonstrates substantial potential, especially within the construction materials sector. Research has demonstrated that fly ash can function as a highly effective supplementary cementitious material in concrete manufacturing [7]. Its incorporation improves concrete’s workability and structural longevity while substantially lowering the carbon emissions associated with traditional cement production [8,9]. Encouraging the full-scale utilization of fly ash can help reduce the environmental burden of coal-based power generation and advance the construction industry’s shift toward sustainability and low-carbon practices [10]. This strategy is consistent with the principles of a circular economy and supports the achievement of carbon neutrality objectives, thereby generating environmental, economic, and long-term industrial benefits.

When fly ash is used to replace cement, the pozzolanic reaction, in which fly ash is involved, and the hydration reaction of cement are particularly important for the compressive strength of fly ash–cement mortar and concrete. In 2018, 12.66 million tons of fly ash were used in Japan, an increase of about 400,000 tons compared with 2017 [11]. Civil engineering accounts for 14.8% and 1.87 million tons of fly ash use, which is the highest use other than for cement. The influence of fly ash on the performance of concrete is largely determined by the characteristics of the source coal and the subsequent combustion and processing conditions. Due to variations in coal types and combustion parameters, the resulting fly ash often exhibits significant variability in key properties such as active component content, fineness, and loss on ignition. Consequently, a considerable portion of the produced fly ash fails to meet the stringent requirements specified by the Japanese Industrial Standards (JIS) [12]. Therefore, to ensure its effective and safe utilization in construction applications, appropriate pretreatment or modification techniques must be applied to enhance the quality of raw fly ash and bring it into compliance with established standards. The main methods for carbon removal from fly ash are electrostatic separation, froth flotation, and gravity separation [13,14]. The flotation method is the most efficient and effective method for removing carbon from fly ash [15,16]. We have developed a flotation machine that can decarbonize fly ash quickly and efficiently [17]. Factors such as the particle size of the modified fly ash, curing temperature, water–ash ratio, and fly ash addition rate affect the cement hydration and pozzolanic reaction [18,19].

The durability and hardening properties of concrete are influenced by the particle size and particle size distribution of fly ash [20]. The calcium hydroxide generated in the hydration reaction is an important reactant in the pozzolanic reaction and thus fly ash–cement mixtures and their concrete have more complex properties compared with standard cement mixtures [21]. The gel products, predominantly composed of calcium silicate hydrate (C-S-H) gel, formed through the pozzolanic reaction can effectively fill the voids within the fly ash–cement matrix, thereby improving the durability of the concrete [22,23]. The particle size of fly ash directly affects the degree of reaction of fly ash, and there is a need to produce finer fly ash.

The properties of fly ash exhibit significant variation due to differences in its source materials and chemical composition, which in turn influence its performance in flotation behavior, pozzolanic reactivity, and application as a cement admixture [24]. According to existing studies, in terms of functional performance, fly ash generated from a specific power plant in Turkey demonstrates relatively high carbon removal efficiency [25]. When the fly ash content from the Deteng Concentrator is below 15%, it can effectively improve the compressive strength of concrete [26]. Both experimental results and predictive models suggest a strong correlation between the apparent diffusion coefficient of fly ash from the United States and its cement substitution rate [27]. In terms of treatment effectiveness, Thai fly ash subjected to air classification technology shows enhanced pozzolanic reactivity. Moreover, the particle size of fly ash has been widely recognized as a critical factor influencing its reactivity and performance in cementitious systems [28]. However, most current research focuses on fly ash derived from a single region or source, with limited systematic comparative studies on fly ash from diverse geographical and compositional origins. This limitation hinders a comprehensive understanding of the cross-regional and cross-type applicability of fly ash.

Testing the reaction rate and calcium hydroxide content in fly ash–cement systems using selective dissolution methods and thermal analysis gives a more accurate assessment of the pozzolanic reaction [29,30]. The selective dissolution method determines the amount of insoluble residue in a sample after reactions with hydrochloric acid and sodium carbonate. The reaction rate is calculated based on the amount of dissolved alumina. Haha et al. [25] tested solvents for determining the pozzolanic reaction using siliceous fly ash and other materials and confirmed the feasibility of the selective dissolution method for calculating the reaction rate. In the chemical reaction of fly ash–cement systems, the cement produces calcium hydroxide during hydration, and the fly ash consumes calcium hydroxide in the pozzolanic reaction. Therefore, thermal analysis is often used to determine the progress of the pozzolanic reaction. Scrivener et al. [31] used thermal analysis to verify the accuracy of the microstructure analysis. Kim et al. [32] used thermal analysis to verify the effect of calcium hydroxide content on the water and the reaction. The chemical composition of fly ash–cement systems at different material ages is often determined by X-ray diffraction (XRD). In XRD experiments, the main chemical compositions tested include C_3_S, C_2_S, C_3_A, and C_4_AF. Among these, the main mineral in cement clinker is C_3_S, which is fired into the raw cement material at 1260–1450 °C and is an important component for analysis.

There are few studies that focus on the replacement of cement with fly ash from different coal sources after modification, classification, and dewatering, in which high amounts of fly ash of different particle sizes are used. The physical and chemical properties, the pozzolanic reaction, and the degree of the hydration reaction are unknown in these materials. Although considerable efforts have been made to study the chemical reactions, most studies have focused on studying fly ash from the same country or region or have used only flotation for fly ash modification. This information is not sufficient to optimize the properties of fly ash.

Based on this study, fly ash from two coal sources was treated using flotation and classification to evaluate its performance and validate the necessity of modification. The treated samples were tested via XRD, thermal analysis, and selective dissolution to assess their physicochemical properties and cementitious performance. The results demonstrate significant source-dependent variations in fly ash behavior and confirm that processing is essential to meet standards and enable high-volume utilization in cement mortar.

## 2. Materials and Methods

### 2.1. Materials

The materials used are shown in Table 1. The cement was Ordinary Portland Cement (OPC) conforming to JIS R 5210. The fly ash samples for blending were collected from two coal sources produced from thermal power plants in Japan. The two fly ash coal sources were from Wambo, Indonesia (hereinafter referred to as W ash), and Ravenworth, Australia (hereinafter referred to as R ash). The fly ash will be processed by flotation, which is the key unit operation in the process, using a lab-scale flotation unit equipped with an integrated size classifier. After flotation, the fly ash was separated by size and dewatered with a special apparatus. The loss on ignition (LOI) was used to determine the unburned carbon content of the samples before and after fly ash flotation. The LOI was calculated using the following Equation (1).

(1) $$\mathrm{LOI}(\%)=\frac{W_{b}-W_{a}}{W_{b}}\times 100$$

*W_b_*: The weights of the samples before burning.
*W_a_*: The weights of the samples after burning.

### 2.2. Fly Ash Modification System

#### 2.2.1. Structure of Fly Ash Modification System

**Figure 1:**
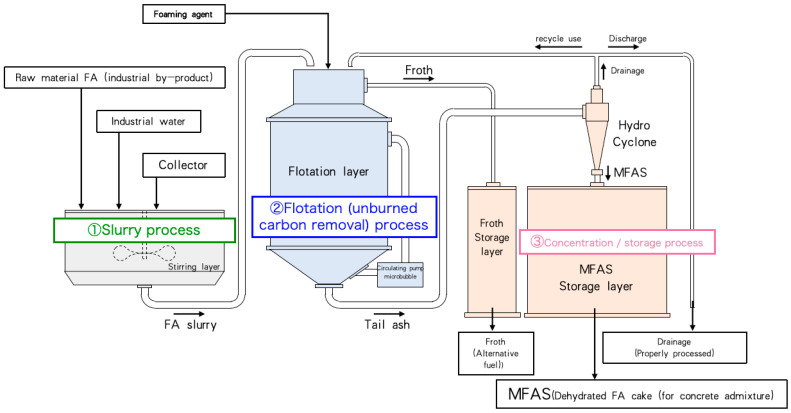
The process of FA modification.

Figure 1 illustrates the fly ash modification process, which was developed to efficiently remove unburned carbon with minimal energy consumption and reduced environmental impact. By operating without external heating, the system significantly lowers processing costs and avoids greenhouse gas emissions typically associated with thermal treatment. Furthermore, it facilitates the production of high-strength, durable low-carbon concrete from low-quality fly ash. The process comprises three main stages: (1) Slurry Preparation: Fly ash is blended with kerosene—serving as a collector to enhance the adhesion of carbon particles to air bubbles—and water. The mixture is agitated in a mixer and subsequently pumped into the flotation beneficiation unit. (2) Flotation Decarbonization: Unburned carbon is removed in a flotation chamber equipped with a circulation pump and a microbubble generator capable of producing bubbles smaller than 100 μm in diameter to improve separation efficiency. Froth enriched with unburned carbon is collected from the top of the unit and discharged, while a modified slurry with reduced carbon content (∼90% moisture) is recovered from the bottom. (3) Concentration and Storage: The slurry is classified and concentrated using a wet cyclone into coarse particles (≤40% moisture) and fine particles (≥90% moisture). The fine slurry is further dewatered into a cake (≤25% moisture) via a decanter centrifuge operating continuously. The separated liquid (≥99% moisture) is either recycled back into the flotation process or treated to comply with wastewater environmental standards.

#### 2.2.2. Classification, Concentration, and Dehydration Experiments

**Figure 2:**
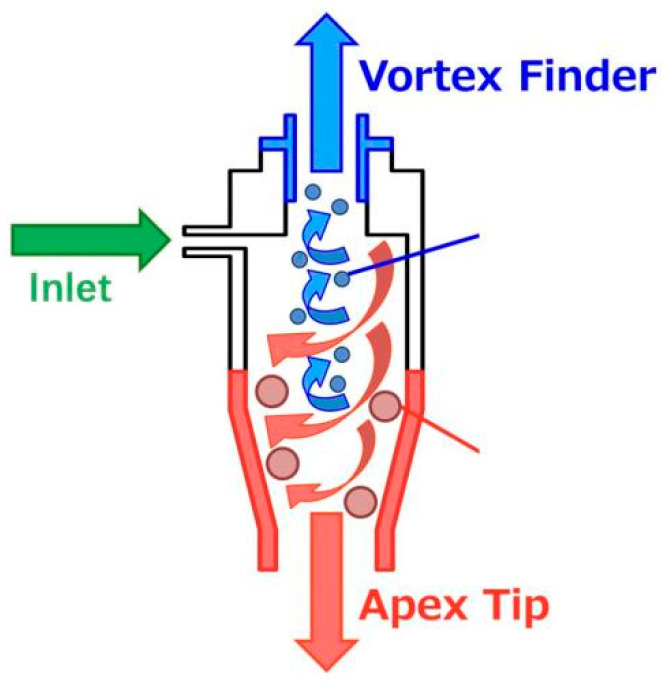
The grading device.

Classification experiments for slurry concentration were performed in a wet cyclone unit, and an additional decanter centrifuge unit was used to perform modified fly ash slurry dewatering experiments. Figure 2 shows the grading device that we used. The classifier/concentrator has two outlets, namely the vortex finder (overflow; OF) and the apex tip (underflow; UF), and can separate the slurry into large and small particles. When the slurry is injected into the liquid cyclone, centrifugal force causes the particles to be arranged in a satellite-like pattern; the specific gravity and particle diameter of the particles toward the periphery are larger, whereas those of particles toward the center are smaller. The larger particles are guided by the flow to the apex tip and are discharged. In contrast, in the center of the cyclone, an upward flow is generated that discharges particles with small specific gravity and particle diameter into the vortex finder. This process separates, classifies, and concentrates the particles in the liquid cyclone.

**Figure 3:**
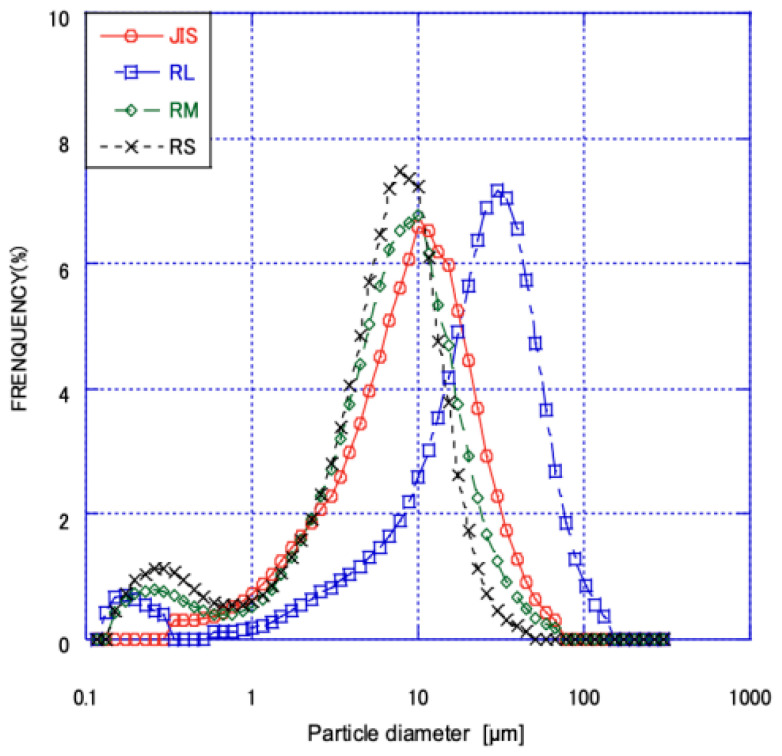

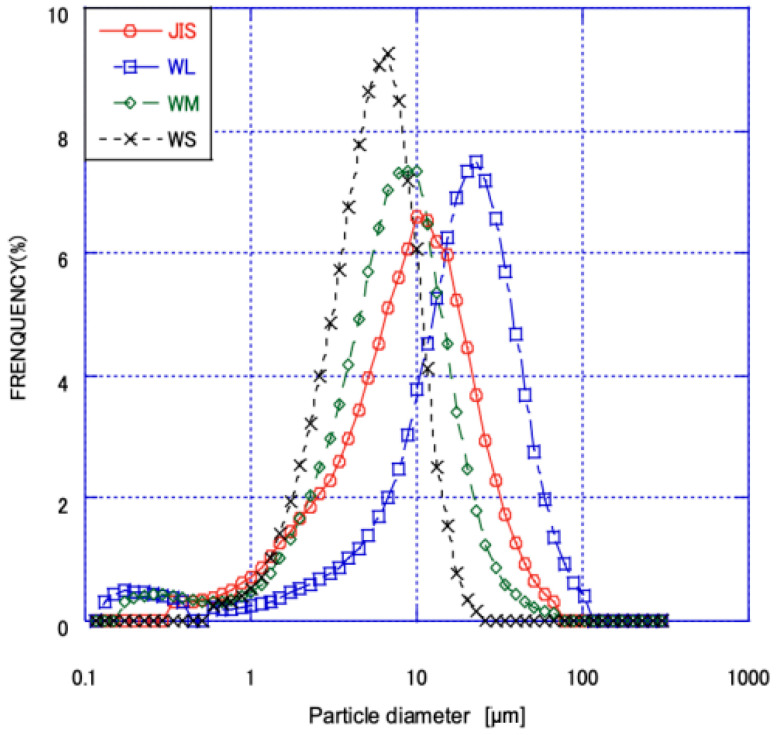
The particle size distribution after grading.

We used this method to classify fly ash from two coal sources into three types: large, small, and unclassified. The fly ash sample name is in the format of a letter denoting the fly ash source (R or S) followed by a letter denoting the size (large: L; small: S; and unclassified: M); for example, WL, WM, and WS. The slurry for RS and WS came from the vortex finder, whereas the slurry for RL and ML came from the apex tip. Unclassified RM and WM ash entered the dewatering system directly after flotation. During the modification process, kerosene (2%) was added as an unburned carbon trapping agent with water and fly ash for mixing, and then the slurry was added to the flotation machine with pine oil (0.2%) as a foaming agent to prepare high-quality modified fly ash. The LOI of fly ash obtained by the flotation method generally complied with JIS specifications (Table 2), although the coarser fraction (WL) from W ash remained above the JIS II limit due to incomplete carbon removal. This fraction accounted for approximately 15% of the total processed W ash. The particle size distribution after grading is shown in Figure 3, and it is compared with that of JIS ash.

### 2.3. Experimental Methods

To test the hydration properties of the cement, we used a high volume of fly ash with a 50% addition ratio (Table 3). The water-to-cement (W/C) and water-to-binder (W/B) ratios, ranging from 0.40 to 0.50, were intentionally selected to accomplish the following: (a) ensure adequate workability of the high-volume fly ash (50% replacement) mortar mixtures, which tend to exhibit higher viscosity, and (b) evaluate the performance under demanding conditions relevant to applications such as flowable fills or mass concrete, where workability is crucial, thereby rigorously assessing the efficacy of the fly ash classification process. The samples were removed at ages of 7, 28, and 91 days and crushed with an automatic grinder. The samples were sieved and powders with particle sizes less than 300 µm were used to test the activity and fluidity of the fly ash, perform microstructure analysis by scanning electron microscopy with energy-dispersive X-ray spectroscopy (SEM-EDS), determine the calcium hydroxide content via thermogravimetric analysis (TGA), quantify the fly ash reaction rate using the selective dissolution method, and measure the unreacted C_3_S content by X-ray diffraction (XRD).

To examine the flow value ratio, mortar made of modified fly ash with different particle sizes and standard sand as aggregate was tested. Mortar was prepared using a standardized mixer according to JIS R5201, followed by calculation using Equation (2) [33]:

(2) $$F=\frac{l_{2}}{l_{1}}\times 100$$

*F*: Flow value ratio.
*l*_2_: Flow value of the base mortar.
*l*_1_: Flow rate value of the samples

The mortar was poured into a steel triple mold and demolded after 1 day. The mortar specimens were cured underwater at 20 °C ± 1 °C with a loading surface of 40 × 40 mm. The activity test was performed at 7, 28, and 91 days according to JIS A 6201 (test method for flow value rate and activity index with mortar). The calcium hydroxide content was determined by TGA [34], following the procedures outlined in the Japanese Manual of Concrete Testing and Analysis. The calcium hydroxide content was tested using the Japanese Manual of Concrete Testing and Analysis as a benchmark. After soaking in acetone for 2 h to stop the hydration reaction and remove free water, it was vacuum dried for 24 h. The powder was then ground and filtered, and only samples less than or equal to 300 μm were used for testing. The weight loss values were calculated near room temperature and at 450 °C with a thermal analyzer to exclude the variation of water binding with the age of the material.

The fly ash reaction rate was quantified using the selective dissolution method [35] followed by centrifugal separation (H-40F, KOKUSAN, Ayabe, Japan). The unreacted amount of C_3_S was determined with a fully automated multifunctional X-ray diffractometer (SmartLab, Rigaku, Akishima, Japan) by Rietveld refinement using analytical software PDXL 2.4 (Rigaku) [36].

## 3. Results and Discussion

### 3.1. Analysis of the Physical Properties of Fly Ash

The LOI of raw ash and reformed and classified fly ash are shown in Table 4. The carbon content was significantly reduced after flotation, with further improvement achieved through classification. Specifically, the LOI of overflow (OF) ash was higher than that of unclassified ash, which can be attributed to the removal of low-density unburned carbon particles via centrifugal force during classification. The results indicate that the carbon content of fly ash was substantially reduced after flotation, but carbon removal was increased by classification. The LOI of OF ash among all ash species was higher than before the scale than before classification. This result was explained by the discharge of unburned carbon with small specific gravity from the OF due to centrifugal force. The fly ash source affected the carbon content, and the overall carbon content of R ash after reforming and classification was much lower than that of W ash compared with the control JIS II fly ash. The carbon content of RS and RM was lower than that of JIS II.

**Figure 4:**
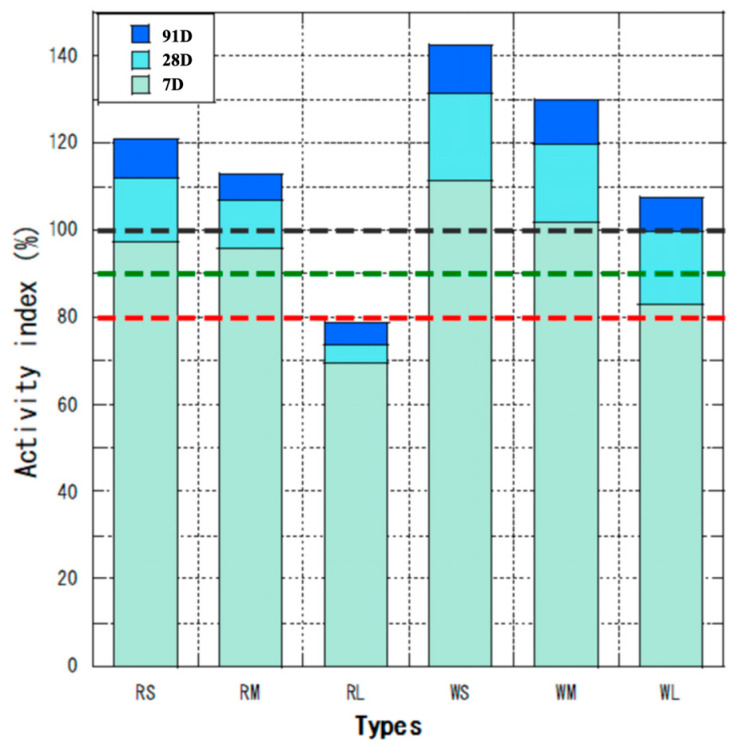
The activity of the fly ashes.

**Figure 5:**
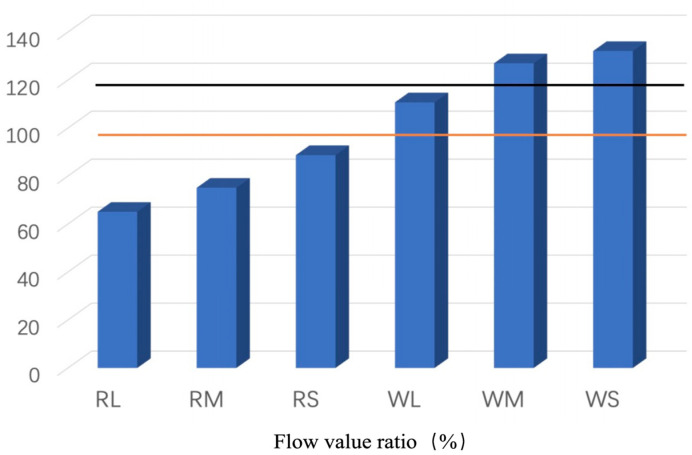
Flow values of the fly ashes.

The activity and flow values of the fly ashes with different particle sizes are shown in Figure 4 and Figure 5. The red line is 80% of the JIS II specification at a material age of 28 days, the green line is 90% at 91 days, and the black line is 100% at 91 days. The activity indices of both W and R ash increased with decreasing particle size at 28 days. Among the classified ashes, the overflow (OF) fractions (RS and WS), which consist of finer particles, exhibited higher activity indices at all ages compared to the underflow (UF) fractions (RL and WL) and unclassified (M) ash. Among the classified ashes, only RL did not reach the JIS II specification. Considering the pozzolanic reaction after 28 days, it is difficult to meet the criterion of “28-day activity index of 80% or more”, as reported using statistics for JIS Class II products (about 450 data) [37]. The RL ash did not meet the JIS II activity index, highlighting a limitation of the classification method for coarse fractions. In practice, such fractions could be further ground or blended with finer ash to achieve compliance, suggesting an area for process optimization in future applications [38]. The superior activity of OF ash is attributed to its finer particle size, which enhances the micro-filler effect between cement and sand particles, as confirmed in previous studies [39,40].

Figure 5 illustrates that the flow value ratio exhibited a trend comparable to that of the activity index, with an increase observed as particle size decreased. The OF ash (RS and WS) exhibited markedly higher flow value ratios compared to UF and unclassified ashes. Several classified fly ash samples surpassed the JIS I specification (black line), indicating that the flotation–classification process contributes to improved mortar properties, which aligns with previous research findings [8]. The beneficiation and classification process exerted a more significant influence on W ash, implying that precise classification can further promote the effective utilization of fly ash. Ferraris et al. [41] associated the enhanced fluidity of finer fly ash with its reduced particle density, which elevates paste content and consequently improves flowability—further substantiating the importance of post-flotation classification.

Fly ash can change the fluidity of concrete compared with cement, and Ferraris et al. [41] concluded that this is because the fly ash particle size affects its particle density. The lower the density of fly ash particles, the more its paste content increases, and the increased paste content in the fixed-weight binder improves the flowability, especially in mixtures containing finer fly ash. This observation also validates the need for classification of fly ash after flotation.

### 3.2. Thermal Analysis

The calcium hydroxide content of control and fly ash mixtures are presented in Table 5. Calcium hydroxide is produced during the hydration process of ordinary Portland cement. The calcium hydroxide content in fly ash–cement slurry is an important part of the pozzolanic ash reaction and should decrease as the reaction progresses. Interestingly, the calcium hydroxide content in most of the fly ash mixes, including the control group, increased despite the expected consumption by the pozzolanic reaction. This is attributed to the high volume (50%) of cement replacement with fly ash, which reduces the total cement content and hence the total amount of CH produced; however, the remaining CH is not fully depleted due to the ongoing and prolonged pozzolanic reaction, particularly in mixes with finer particles. This observation was consistent with the results of previous studies [42,43,44]. The relationship between particle size and CH content was evident: classified fine ash (OF fractions, e.g., RS and WS) consistently showed lower CH contents at later ages compared to unclassified (M) or coarse (UF) fractions, indicating higher pozzolanic consumption. For instance, at 91 days, WS50 and RS50 exhibited lower CH contents (12.8% and 13.6%, respectively) than their unclassified counterparts (WM50: 11.5%; RM50: 14.6%). This suggests that finer particles provide more surface area for reaction, leading to greater CH consumption.

**Figure 6:**
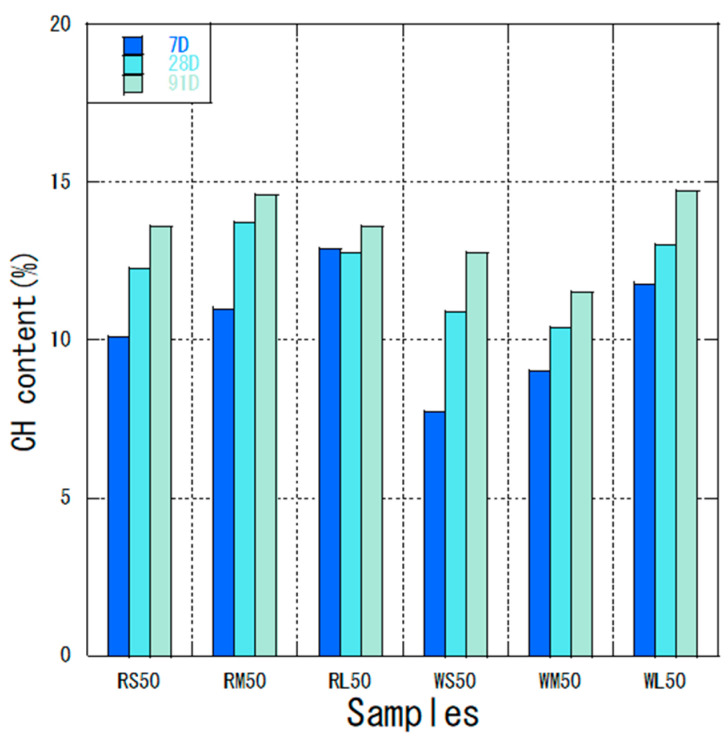
The Ca (OH)_2_ content of fly ash and cement mixture.

Figure 6 shows the calcium hydroxide content of fly ash from different coal sources with different particle sizes at the same water–binder ratio. At 7 days, the initial calcium hydroxide content of W ash with better fly ash activity was lower than that of R ash for different particle sizes. The pozzolanic reaction and other properties of fly ash from different coal sources differed, and the calcium hydroxide content was fundamentally related to the reaction rate, curing temperature, and chemical composition [45]. The calcium hydroxide content of unclassified RM ash was 13.7% and that of WM ash was 10.4% at 28 days. The variation of calcium hydroxide in RM and WM at 91 days was close to 0.9% and 1.1%, respectively. For the classified fly ash–cement mixtures, the variation values of calcium hydroxide content at 91 days were higher for both RS50 and WS50 than for the unclassified fly ash–cement mixtures. This is also consistent with the fluidity of fly ash, where more fluid fly ash continues to react in the late stage of the pozzolanic reaction. The initial calcium hydroxide content of RS50 was much higher than that of WS50 at 7 days, which was attributed to the different mullite content of fly ash from different coal sources, as quantitatively confirmed in Section 3.4 (R ash: 22.5%; W ash: 15.3%). The reaction rate of fly ash is affected by the temperature, the amount of chemically bound water, and the content of less-reactive crystalline phases like mullite [46]. Wang et al. [45] found that mullite affects the reaction rate of fly ash, supporting our findings. The higher mullite content in R ash likely contributed to a slightly slower initial pozzolanic reaction, leading to higher early CH content in RS50 compared to WS50. Therefore, in combination with the characteristics of fly ash, using high volumes of classified fly ash can promote the pozzolanic reaction in the middle and late stages, thus improving the concrete’s durability. Although the absolute reduction in Ca (OH)_2_ content may appear modest at 28 days, it is important to note that in high-volume fly ash systems, even a slight decrease indicates ongoing pozzolanic reactivity, which continues to develop significantly beyond 91 days, as supported by the reaction rate data. Among all fly ash types, the classified fine fractions (WS and RS) showed the most significant consumption of CH at later ages, underscoring their superior pozzolanic reactivity compared to unclassified or coarser fractions.

### 3.3. Reaction Rate

**Figure 7:**
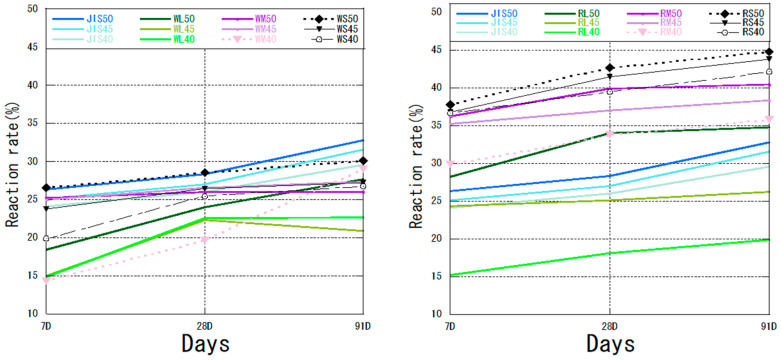
The reaction rate of fly ash and cement mixture.

The reaction rates of fly ash–cement mixtures with different particle sizes are shown in Figure 7. The reaction rates for all W ash mixtures fell within ±5% of the values for the control JIS ash across all curing ages, indicating comparable reactivity. In contrast, the R ash mixtures exhibited a distinct trend, with reaction rates deviating by up to 15% from the control, underscoring the significant influence of coal source on pozzolanic reactivity. The mixture of WS ash and cement was closest to JIS50 at the initial material age at a water–binder ratio of 50%. The reaction rate of WS50 was still the highest of all the W ash mixtures at 91 days, except for JIS ash. This result is consistent with the results for calcium hydroxide content. The overall trend for R ash was similar to that for W ash, and the reaction rate of RS50 at all material ages was higher than that of other samples, including all JIS samples. At day 91, the reaction rate of RS50 was 44.84%. Larger particle sizes correlated with higher LOI, indicating greater unburned carbon content, which inhibits reactivity. Furthermore, coarser particles provide less specific surface area, reducing their availability for dissolution and pozzolanic reactions. As the particle size of fly ash increased, the LOI of fly ash increased, and carbon removal decreased. The reaction rates of the mixtures of fly ash and cement with different coal sources decreased with increasing particle size throughout the pozzolanic reaction. The reaction rate of fly ash with the same particle size decreased as the water–binder ratio decreased. Fly ash, which is an additive to the pozzolanic reaction, is used to fill the void structure through the hydration products generated by its own reaction [47]. Therefore, the particle size of fly ash is particularly important. The reaction rates of unclassified WM and RM ashes at different water–cement ratios had a lower reaction rate increase at the later stage. The reaction rates of RL ash mixtures were generally lower than those of RM and RS ash mixtures, although the reaction rates did not decrease in the later stages, further demonstrating the importance of fly ash classification. Among all mixes, the classified fine fly ash fractions—RS50 and WS50—demonstrated the highest reaction rates across all curing ages, underscoring the critical role of particle size and classification in enhancing pozzolanic reactivity.

**Figure 8:**
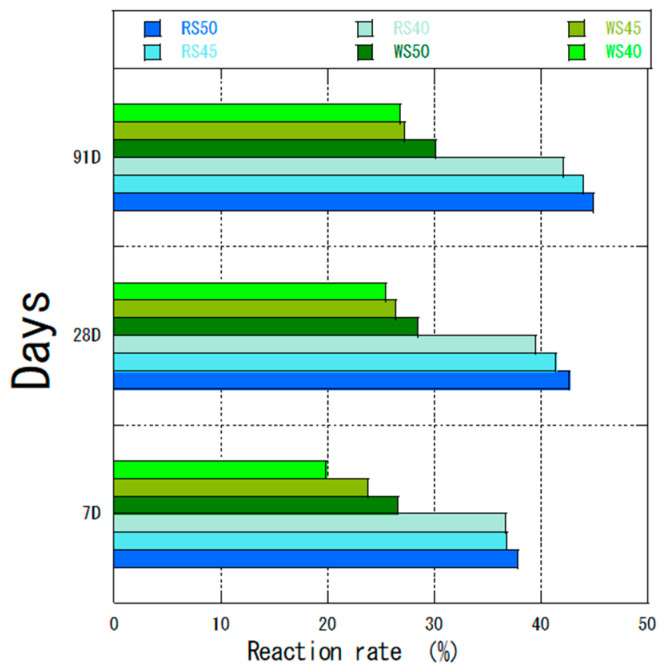
The reaction rate of RS ash– and WS ash–cement mixtures.

Different water–binder ratios were used to test the performance of fly ash. Figure 8 shows the reaction rates of OF ash for W ash and R ash for a water–binder ratio of 50%. The LOI of raw R ash was lower than that of raw W ash (Table 1), although the same trend was observed after flotation classification (Table 2). The LOI of RS ash was 1.1% and that of WS ash was 1.5%. However, the reaction rate of RS ash was higher than that of WS ash at any material age at the same water–binder ratio. Therefore, the content of C_3_S was also an important factor affecting the pozzolanic reaction for fly ash from different coal sources (Table 6). Abdul-Maula et al. [48] concluded that in the middle and late stages of the reaction, the amount of reacted alite in the cement with the addition of fly ash was higher than that in normal Portland cement. This result was consistent with our results for a high dose of fly ash. The overall calcium hydroxide content of WS ash was lower than that of RS ash (Table 6), which also contributed to the lower volcanic ash reaction rate for WS.

The experimental results showed the feasibility of using high volumes of fly ash. For a water–binder ratio of 50%, the high volume of fly ash with a smaller particle size promoted the cement hydration, which was consistent with previous studies [49,50]. For fly ash from different coal sources, sorting and flotation classification can make fly ash suitable for different conditions (e.g., different curing temperatures, etc.) [51]. Thus, the role of fly ash in the hydration reaction and pozzolanic reaction can be promoted to maximize and improve the reaction rate of concrete in the later stages.

### 3.4. X-Ray Diffraction Analysis

The C_3_S contents of R ash– and W ash–cement mixtures at a water–binder ratio of 50% determined by XRD of raw fly ash and its cement mixtures are shown in Table 6. To verify the performance of a high volume of fly ash in the cement system, the fly ash addition rate in the fly ash–cement mixture was 50%. At a water–binder ratio of 50%, the reaction rate and calcium hydroxide content were different for classified fly ashes from different coal sources. Furthermore, the predominant crystalline phases in both ashes were quartz (SiO_2_) and mullite (3Al_2_O_3_·2SiO_2_). The R ash exhibited a significantly higher mullite content (22.5 wt%) compared to the W ash (15.3 wt%). Conversely, the amorphous content, which is crucial for pozzolanic activity, was higher in W ash (68.4 wt%) than in R ash (61.8 wt%). This disparity in phase composition, particularly the higher crystalline mullite content in R ash, is a key factor influencing the reaction kinetics and calcium hydroxide consumption patterns observed in the cement mixtures. The consumption of C_3_S, a primary mineral in cement clinker, serves as a key indicator of the degree of cement hydration. Its decline reflects the progression of both hydration and pozzolanic reactions. Although the initial C_3_S content of the course WL50 fraction (14.69% at 7 days) was comparable to that of the R ash mixtures, its subsequent hydration behavior aligned with other W ash mixtures, exhibiting a faster consumption rate compared to R ash mixtures of similar particle size (e.g., RL50). The C_3_S content in the R ash–cement mixture was higher than that in the W ash–cement mixture in the first and middle stages [52]. C_3_S reacts early in the hydration process; thus, its content decreases sharply during initial stages. RS50 showed a 10.36% decrease in C_3_S content at 28 days, and WS50 showed a smaller decrease of 7.43%. At 91 days, the decreases for RS50 and WS50 were 4.07% and 2.98%. Even at the late stage of hydration, the hydration of R ash was still higher than that of W ash. This result was consistent with the calcium hydroxide content and reaction rate; the calcium hydroxide content of RS50 was higher than that of WS50 in the late stage of pozzolanic reaction, and the reaction rate was also higher. The calcium hydroxide content was still increasing at the late stage because of the high volume of fly ash used. The products of the pozzolanic reaction accumulated in the voids of the mixture, resulting in the slow reaction of most of the generated calcium hydroxide, which could further increase the durability of the concrete at the later stage [53,54]. The higher C_3_S content in the R ash–cement mixtures resulted in higher reaction rates than both W ash–cement mixtures. This result was also consistent with previous studies [44].

The performance of the various fly ash–cement mixtures after classification also varied. The difference in C_3_S content between RS50 and RL50 at the early stage of the pozzolanic reaction was 1.87%, and the difference between WS50 and WL50 was 3.81%. As C_3_S was consumed by the hydration reaction, the rate of change in C_3_S content in the mixtures of different particle sizes gradually decreased in the middle and later stages of the reaction. The initial C_3_S content of WL50 was higher than that of RL50 at 91 days, and the C_3_S content of the same type of fly ash increased with the increase in particle size in the same stage. The higher residual C_3_S content in WL50 and RL50 indicates that larger particles hydrate more slowly and may remain underutilized within 91 days. This suggests that while coarser fractions can contribute to long-term strength, their reactivity is limited within typical curing periods, underscoring the importance of particle size optimization. This pattern confirms that finer classified ash (OF fractions, e.g., RS50 and WS50) consistently exhibited greater C_3_S consumption across all ages, indicating higher hydration and pozzolanic activity compared to coarser or unclassified fractions. This indicates that larger particle sizes are less favorable for the hydration and pozzolanic reactions. Thus, the particle size affects the performance of concrete after fly ash is added. Zeng et al. [47] simulated the degree of cement hydration in a model and calculated the degree of reaction of fly ash particulate matter in the mixture slurry. Their results were consistent with our experimental results. In contrast, the fine particles in the mixture of RM50 and WM50, where the ash was not classified, reacted preferentially as the reaction proceeded. The fine particles were consumed by the reaction, but the coarse particles only started to react gradually [55]. Therefore, RS50 and WS50 with a high fine particle content after classification had a better reaction performance.

## 4. Conclusions

The effects of fly ash particle size and coal source on the performance of cement systems were investigated through a novel flotation–classification approach. The physical properties of fly ash were measured to reveal the variability between different particle sizes of fly ash from different coal sources. Fly ash–cement mixtures with different water-to-ash ratios were tested using thermal analysis to analyze the effect of their calcium hydroxide content on the cement system. The reaction rates of the mixtures were further tested using the selective dissolution method. Samples of different particle sizes were selected for analysis after x-ray diffraction experiments. Future studies could benefit from the integration of computer vision techniques for automated particle size and morphology analysis. For instance, DeepLab (Chen et al., 2020) offers semantic segmentation capabilities for precise particle boundary detection, while EfficientNet (Tan & Le, 2024) provides efficient and scalable image classification [56,57]. These methods could enable high-throughput, consistent characterization of fly ash, facilitating more robust classification and quality control. The main results of this study are summarized as follows:

(1) Physical Properties: After processing, W ash generally exhibited higher activity and flow values compared to R ash. In particular, the classified fine fraction of W ash (WS) surpassed the specifications of JIS Class I fly ash, demonstrating that the flotation-classification process significantly enhances fly ash properties.
(2) Reactivity: The use of high-volume fly ash (50% replacement) promoted prolonged pozzolanic reaction, especially with finer particles, as indicated by the continuous consumption of calcium hydroxide at later ages. Smaller particle sizes led to greater decreases in CH content, highlighting their enhanced reactivity.
(3) Cement Hydration and Performance: Finer fly ash particles consistently resulted in higher consumption of C_3_S across all ages, indicating accelerated cement hydration and pozzolanic activity. The overall performance of R ash was superior to W ash, attributable to its inherent mineral composition. Classification significantly improved reactivity, with overflow (OF) fractions showing the highest reaction rates and pozzolanic consumption. While smaller particle sizes generally enhance pozzolanic reactivity, the plateauing of reaction rates in coarser fractions (e.g., RL) suggests that chemical and mineralogical factors also impose limitations. Thus, both particle size and phase composition must be considered to fully optimize fly ash performance.

## Figures and Tables

**Table 1:** Comparative numerical analysis of material constituents.

Symbol		Type	Physical Characteristics
Cement	C	Portland cement	Density: 3.16 g/cm^3^
Admixture	FA	Raw ash fly ash (W ash)	Density: 2.19 g/cm^3^ Specific surface area: 6810 g/cm^3^ Loss on ignition: 23.5% PH: 6.98%
		Raw ash fly ash (R ash)	Density: 2.19 g/cm^3^ Specific surface area: 5500 g/cm^3^ Loss on ignition: 8.5% PH: 4.34%
Water	W	Tap water	-

**Table 2:** LOI of fly ash before and after modification.

Types		LOI (%)
Raw fly ash	R ash	8.5
Modified fly ash	RS	1.1
	RM	1.2
	RL	2.8
Raw fly ash	W ash	24.8
Modified fly ash	WS	1.5
	WM	1.8
	WL	3.9

**Table 3:** Experiment formulation.

Type	W/C	W/B	Unit Weight (kg/m^3^)		
			W	C	FA
R/W40	0.80	0.40	180	225	225
R/W45	0.90	0.45	180	200	200
R/W50	1.00	0.50	180	180	180

**Table 4:** LOI for all fly ash.

Types		LOI (%)
Raw fly ash	R ash	8.5
Modified fly ash	RS	1.1
	RM	1.2
	RL	2.8
Raw fly ash	W ash	24.8
Modified fly ash	WS	1.5
	WM	1.8
	WL	3.9
JIS II		1.3

**Table 5:** The Ca (OH)_2_ content of control and fly ash mixtures.

Sample	7D	28D	91D
JIS40	10.4	10.3	13.1
RL40	9.6	13.4	13.9
RM40	11.2	14.9	13.9
RS40	9.0	10.9	12.7
WL40	9.4	13.0	13.3
WM40	8.2	11.2	11.6
WS40	7.9	9.5	11.2
JIS45	11.0	12.9	15.4
RL45	11.1	15.4	16.5
RM45	11.2	10.8	13.8
RS45	9.5	10.7	13.5
WL45	11.5	14.4	14.7
WM45	9.1	11.1	11.7
WS45	9.7	11.9	11.0
JIS50	11.2	12.5	15.0
RL50	12.9	12.8	13.6
RM50	11.0	13.7	14.6
RS50	10.1	12.3	13.6
WL50	11.8	13.0	14.7
WM50	9.0	10.4	11.5
WS50	7.7	10.9	12.8

**Table 6:** The C_3_S content of different fly ash–cement mixtures.

Types	C_3_S (%)		
	7D	28D	91D
RS50	14.92	4.56	0.49
RM50	15.42	5.01	0.75
RL50	16.79	5.42	1
WS50	10.88	3.45	0.47
WM50	11.65	3.99	0.68
WL50	14.69	5.02	1.01

## Data Availability

The data presented in this study are available on request from the corresponding author. The data are not publicly available due to privacy restrictions.
